# Association between hypertension requiring medication and postoperative 30-day mortality in adult patients with tumor craniotomy: an analysis of data using propensity score matching

**DOI:** 10.3389/fneur.2024.1412471

**Published:** 2024-09-17

**Authors:** Yufei Liu, Haofei Hu, Wenjian Zheng, Zhong Deng, Jihu Yang, Xiejun Zhang, Zongyang Li, Lei Chen, Fanfan Chen, Nan Ji, Guodong Huang

**Affiliations:** ^1^Department of Neurosurgery, Shenzhen Key Laboratory of Neurosurgery, Shenzhen Second People’s Hospital, The First Affiliated Hospital of Shenzhen University, Shenzhen, China; ^2^Nephrological Department, Shenzhen Second People’s Hospital, The First Affiliated Hospital of Shenzhen University, Shenzhen, China; ^3^Neurosurgical Department, Beijing Tiantan Hospital, Capital Medical University, Beijing, China

**Keywords:** brain tumor, craniotomy, hypertension, propensity score matching, mortality

## Abstract

**Background:**

Reliable quantification of the association between hypertension requiring medication and postoperative 30-day mortality in adult patients who undergo craniotomy for tumor resection is limited. We aimed to explore the associations between these factors.

**Materials and methods:**

This work was a retrospective cohort study that used propensity score matching (PSM) among 18,642 participants from the American College of Surgeons National Surgical Quality Improvement Program database between 2012 and 2015. Hypertension requiring medication and postoperative 30-day mortality were the independent and dependent target variables, respectively. PSM was conducted via nonparsimonious multivariate logistic regression to balance the confounders. Robust estimation methods were used to investigate the association between hypertension requiring medication and postoperative 30-day mortality.

**Results:**

A total of 18,642 participants (52.6% male and 47.4% female) met our inclusion criteria; 7,116 (38.17%) participants with hypertension required medication and had a 3.74% mortality rate versus an overall mortality rate of 2.46% in the adult cohort of patients who underwent craniotomy for tumor resection. In the PSM cohort, the risk of postoperative 30-day mortality significantly increased by 39.0% among patients with hypertension who required medication (OR = 1.390, 95% confidence interval (CI): 1.071–1.804, *p* = 0.01324) after adjusting for the full covariates. Compared with participants without hypertension requiring medication, those with hypertension requiring medication had a 34.0% greater risk of postoperative 30-day mortality after adjusting for the propensity score (OR = 1.340, 95% CI: 1.040–1.727, *p* = 0.02366) and a 37.6% greater risk of postoperative 30-day mortality in the inverse probability of treatment weights (IPTW) cohort (OR = 1.376, 95% CI: 1.202, 1.576, *p* < 0.00001).

**Conclusion:**

Among U.S. adult patients undergoing craniotomy for tumor resection, hypertension requiring medication is a notable contributor to 30-day mortality after surgery, with odds ratios ranging from 1.34 to 1.39.

## 1 Background

Craniotomy, the fundamental surgical procedure for treating the vast majority of brain tumors, is associated with significant morbidity and mortality (1, 2). Postoperative 30-day mortality, which is a significant measure of mortality during the postoperative period, offers a reliable assessment of the safety of surgeries and the likelihood of postoperative complications in patients who undergo noncardiac procedures, including craniotomy (2–5). Hypertension, a rapidly increasing worldwide public health issue, is the primary contributor to cardiovascular disease and mortality (6–8).

Hypertension has been acknowledged as a risk factor for postoperative complications and/or mortality in numerous surgeries (9–14). Severe perioperative hypertension can result in excessive surgical bleeding, congestive heart failure, myocardial infarction and acute pulmonary oedema (15). A preoperative systolic blood pressure above 120 mmHg is associated with an increased mortality hazard ratio in cancer patients (16). Hypertension requiring medication was identified as a risk factor for complications occurring within 30 days after surgery in head and neck free tissue transfer (9). Hypertension significantly increases the incidence of postoperative complications during craniotomy for meningioma patients (11). In addition to being a predictor of reoperation for haematoma after craniotomy for tumors based on the analysis of the National Surgical Quality Improvement Program (ACS NSQIP) (12), hypertension was also found to be linked to greater cardiac complications in patients who underwent carotid endarterectomy (13). Hypertension requiring medication was recorded in the ACS NSQIP database. Limited research has been conducted on the correlation between hypertension requiring medication and postoperative 30-day mortality after craniotomy for tumor resection in adult patients. The traditional parsimonious regression model used in previous studies could result in biases caused by remaining or unmeasured confounding factors or overfitting the model (17), which could hinder the detection of the link between hypertension requiring medication and postoperative 30-day mortality. However, propensity score matching (PSM) was used to adjust for heterogeneity in the baseline characteristics (18–20). Hence, conducting a comprehensive retrospective study utilizing PSM data is imperative to assess the correlation between hypertension requiring medication and postoperative 30-day mortality. This study utilized real-world data from a sample of 18,642 adult patients who underwent craniotomy for tumor resection between 2012 and 2015.

## 2 Participants and methods

### 2.1 The source of data

The data were extracted from the ACS NSQIP for the present retrospective cross-sectional study. We freely obtained the raw data labeled [S1 Data] from Zhang et al.’s publication titled “Sepsis and Septic shock after craniotomy: Predicting significant patient safety and quality outcome measure.” The article can be accessed at the following website: https://www.ncbi.nlm.nih.gov/pmc/articles/PMC7498000/. The initial study included a total of 18,642 individuals who underwent craniotomy for brain tumors between 2012 and 2015. This research was published as an article available to the public, allowing unrestricted distribution, use, and reproduction under the Creative Commons Attribution License. The study was conducted at approximately 400 academic and community hospitals throughout the United States. Hence, our research utilized the provided dataset consisting of 18,642 individuals to conduct a secondary analysis without infringing upon the original authors’ rights.

### 2.2 Data collection

The data collected in our study included the following variables: (1) continuous variables such as height, weight, and preoperative blood test indicators (hematocrit (HCT), sodium (Na), blood urea nitrogen (BUN), white blood cell (WBC) count, creatine (Cr), and platelet (PLT) count), as well as operating time; and (2) categorical variables, including sex, race, age range, diabetes status, smoking status, year of operation, functional health status, severe chronic obstructive pulmonary disease (COPD), congestive heart failure (CHF), hypertension requiring medication, preoperative transfusions, disseminated cancer, preoperative systemic sepsis, steroid use for chronic conditions, >10% loss of body weight in the last 6 months, bleeding disorders, emergency cases, and wound classification. More elaborate details were provided in the original study. Body mass index (BMI) (kg/m^2^) was calculated by dividing the square of height in meters by weight in kilograms. The data were collected in accordance with standardized conditions and processed via consistent procedures. Our study received exemption from the Clinical Research Ethics Committee of our center.

### 2.3 Outcome measures

The focus is on the result of postoperative 30-day mortality, which refers to the occurrence of death within the initial 30 days following discharge from surgery. The variables of interest and the factors that May influence them were investigated. The exposure of interest was hypertension requiring medication. Typically, an individual is considered to have hypertension if their blood pressure is consistently at or above 140/90 mmHg. To be considered for surgery, the patient’s medical record must show that they have documented hypertension and that it is sufficiently severe to necessitate the use of antihypertensive medication (such as diuretics, beta blockers, angiotensin-converting enzyme (ACE) inhibitors, and calcium channel blockers) within 30 days before the main operative procedure or when they are being evaluated as potential candidates for surgery. Hypertension requiring medication was recorded as a categorical variable (yes/no). Patients without hypertension requiring medication were defined as patients who did not have high blood pressure or who had high blood pressure but did not have medication to control it before surgery.

### 2.4 Statistical analyses

The mean (standard deviation) or median (range) was used to express continuous variables with a normal or nonnormal distribution, respectively, whereas categorical variables were represented as the No. (%). To examine distinctions among various groups, we employed the t test (assuming a normal distribution), the chi-square test (for categorical variables), or Wilcoxon’s rank sum test (for skewed distributions). There were 730 patients (3.92%) with missing BMI values (weight and/or height), 1,532 patients (8.22%) with missing BUN values, 709 patients (3.8%) with missing Cr values, 592 patients (3.18%) with missing WBC values, 440 patients (2.36%) with missing HCT values, and 579 patients (3.11%) with missing PLT values. To address the missing data of covariants, multiple imputations were employed. The analysis of missing data utilized the assumption of missingness at random (MAR).

Creating a well-balanced group via PSM May help minimize notable disparities between patients with hypertension requiring medication and those without it (Table 1). For hypertension requiring medication, the PS was determined by employing an extensive multivariate logistic regression model, wherein hypertension served as the outcome variable and all other initial attributes were considered covariates (21). Table 1 shows the covariates used in the model. The matching variables consisted of BMI, HCT, Na, BUN, WBC, Cr, PLT, duration of surgery, sex, ethnicity, age group, presence of diabetes, smoking habits, year of surgery, functional health status, COPD, CHF, presurgical transfusions, disseminated cancer, presurgical systemic sepsis, use of steroids for chronic conditions, weight loss exceeding 10% in the last 6 months, bleeding disorders, emergency cases, and wound classification. The PSM utilized a 1:1 greedy algorithm that did not involve replacement and had a caliper width of 0.01. In addition, we attempted to locate a stricter caliper; however, the model that provided the closest match was 0.01. To assess the degree of balance between the matched groups, we calculated the standardized difference (SD) for each covariate at the beginning of the study. Satisfaction with the matching was considered acceptable if the standardized mean differences were less than 10% (22, 23). The C-index was computed for the logistic regression model employed in propensity score matching.

**Table 1:** Baseline characteristics before and after propensity score matching in the original cohort.

Characteristic	Before matching			After matching		
Participants	Nonhypertension	Hypertension	SD (100%)	Nonhypertension	Hypertension	SD (100%)
*N*	11,526	7,116		5,099	5,099	
BMI	27.667 ± 6.367	30.283 ± 6.998	39.1	29.207 ± 7.117	29.354 ± 6.412	2.2
Na	138.781 ± 3.063	138.347 ± 3.461	13.3	138.574 ± 3.205	138.534 ± 3.378	1.2
BUN	15.000 (11.204–19.000)	18.000 (14.000–24.000)	47.5	17.000 (13.000–21.182)	17.000 (13.000–22.000)	2.6
Cr	0.800 (0.679–0.910)	0.860 (0.700–1.030)	26.8	0.800 (0.700–0.976)	0.820 (0.700–1.000)	3.6
WBC	8.200 (6.200–11.300)	8.815 (6.700–12.100)	12.7	8.600 (6.400–11.805)	8.700 (6.600–11.814)	1.5
HCT	40.627 ± 4.654	39.925 ± 5.033	14.5	40.435 ± 4.816	40.340 ± 4.803	2.0
PLT	246.295 ± 75.668	240.181 ± 78.185	7.9	243.893 ± 78.565	242.576 ± 76.790	1.7
Operating time	185.000 (123.000–276.000)	176.000 (118.000–256.000)	9.5	177.000 (117.000–262.000)	177.000 (119.000–259.500)	0.6
Sex			9.3			0.2
Male	5,258 (45.619%)	3,578 (50.281%)		2,464 (48.323%)	2,470 (48.441%)	
Female	6,268 (54.381%)	3,538 (49.719%)		2,635 (51.677%)	2,629 (51.559%)	
Race			19.3			19.3
White Asian	8,252 (71.595%)	5,038 (70.798%)		3,693 (72.426%)	3,565 (69.916%)	
Black or African	354 (3.071%)	189 (2.656%)		122 (2.393%)	148 (2.903%)	
American	565 (4.902%)	680 (9.556%)		254 (4.981%)	497 (9.747%)	
Unknown	2,355 (20.432%)	1,209 (16.990%)		1,030 (20.200%)	889 (17.435%)	
Age ranges (years)			88.4			6.3
18–40	2,825 (24.510%)	232 (3.260%)		287 (5.629%)	226 (4.432%)	
41–60	5,405 (46.894%)	2,337 (32.841%)		1979 (38.812%)	2081 (40.812%)	
61–80	3,131 (27.165%)	4,102 (57.645%)		2,668 (52.324%)	2,635 (51.677%)	
>81	165 (1.432%)	445 (6.254%)		165 (3.236%)	157 (3.079%)	
Diabetes, *N* (%)			55.7			14.0
No	10,994 (95.384%)	5,468 (76.841%)		4,616 (90.528%)	4,456 (87.390%)	
Yes (Noninsulin)	336 (2.915%)	1,043 (14.657%)		292 (5.727%)	478 (9.374%)	
Yes (Insulin)	196 (1.701%)	605 (8.502%)		191 (3.746%)	165 (3.236%)	
Smoking status, N (%)			6.4			1.4
No	9,184 (79.681%)	5,848 (82.181%)		4,093 (80.271%)	4,122 (80.839%)	
Yes	2,342 (20.319%)	1,268 (17.819%)		1,006 (19.729%)	977 (19.161%)	
Functional health status			12.5			0.8
Independent	11,161 (96.833%)	6,710 (94.295%)		4,877 (95.646%)	4,871 (95.529%)	
Partially dependent	327 (2.837%)	350 (4.918%)		193 (3.785%)	196 (3.844%)	
Totally dependent	38 (0.330%)	56 (0.787%)		29 (0.569%)	32 (0.628%)	
Severe COPD, *N* (%)			18.9			1.5
No	11,190 (97.085%)	6,617 (92.988%)		4,839 (94.901%)	4,822 (94.568%)	
Yes	336 (2.915%)	499 (7.012%)		260 (5.099%)	277 (5.432%)	
Congestive heart failure, *N* (%)			9.0			0.5
No	11,514 (99.896%)	7,069 (99.340%)		5,090 (99.823%)	5,089 (99.804%)	
Yes	12 (0.104%)	47 (0.660%)		9 (0.177%)	10 (0.196%)	
Disseminated cancer, *N* (%)			8.7			1.4
No	9,197 (79.794%)	5,423 (76.209%)		3,928 (77.035%)	3,898 (76.446%)	
Yes	2,329 (20.206%)	1,693 (23.791%)		1,171 (22.965%)	1,201 (23.554%)	
Steroid use for a chronic condition, *N* (%)			2.7			0.1
No	9,838 (85.355%)	6,005 (84.387%)		4,300 (84.330%)	4,299 (84.311%)	
Yes	1,688 (14.645%)	1,111 (15.613%)		799 (15.670%)	800 (15.689%)	
>10% loss body weight in last 6 months			3.1			1.5
No	11,296 (98.005%)	6,941 (97.541%)		4,993 (97.921%)	4,982 (97.705%)	
Yes	230 (1.995%)	175 (2.459%)		106 (2.079%)	117 (2.295%)	
Bleeding disorders			10.2			1.1
No	11,361 (98.568%)	6,908 (97.077%)		4,989 (97.843%)	4,997 (98.000%)	
Yes	165 (1.432%)	208 (2.923%)		110 (2.157%)	102 (2.000%)	
Preoperative transfusions			4.5			1.4
No	11,499 (99.766%)	7,080 (99.494%)		5,084 (99.706%)	5,080 (99.627%)	
Yes	27 (0.234%)	36 (0.506%)		15 (0.294%)	19 (0.373%)	
Preoperative systemic sepsis, *N* (%)			5.0			0.9
No	11,152 (96.755%)	6,818 (95.812%)		4,918 (96.450%)	4,909 (96.274%)	
Yes	374 (3.245%)	298 (4.188%)		181 (3.550%)	190 (3.726%)	
Emergency case			3.6			0.8
No	10,747 (93.241%)	6,697 (94.112%)		4,753 (93.214%)	4,763 (93.410%)	
Yes	779 (6.759%)	419 (5.888%)		346 (6.786%)	336 (6.590%)	
Wound classification			2.8			1.8
Clean	11,228 (97.415%)	6,899 (96.951%)		4,946 (96.999%)	4,961 (97.294%)	
Nonclean	298 (2.585%)	217 (3.049%)		153 (3.001%)	138 (2.706%)	

To determine the independent relationships between hypertension requiring medication and postoperative 30-day mortality, the researchers utilized the doubly robust estimation technique, which combines a multivariate regression model with a propensity score model. In sensitivity analyses, the inverse probability of treatment weight (IPTW) served as the weight for the estimated propensity score, 1/PS, for individuals with hypertension requiring medication and the inverse of 1 minus the propensity score, 1/(1–PS), for individuals without hypertension requiring medication (24). The IPTW was used to calculate a standardized effect estimate using all study participants as the reference population. In the sensitivity analysis, we included two additional association inference models in both the original cohort and the weighted cohort. The reported and compared effect sizes and *p* values were calculated from all of these models. All the results were reported based on the STROCSS statement (25). Furthermore, we investigated the possibility of unquantified confounding effects between hypertension requiring medication and the likelihood of postoperative 30-day mortality through the computation of E values (26).

Both Empower Stats (X & Y Solutions, Boston, MA, United States) and the statistical package R^1^ were utilized for the statistical analysis. We calculated ORs and 95% CIs. A significance level of less than 0.05 was used to indicate statistical significance.

## 3 Results

### 3.1 Study population

**Figure 1:**
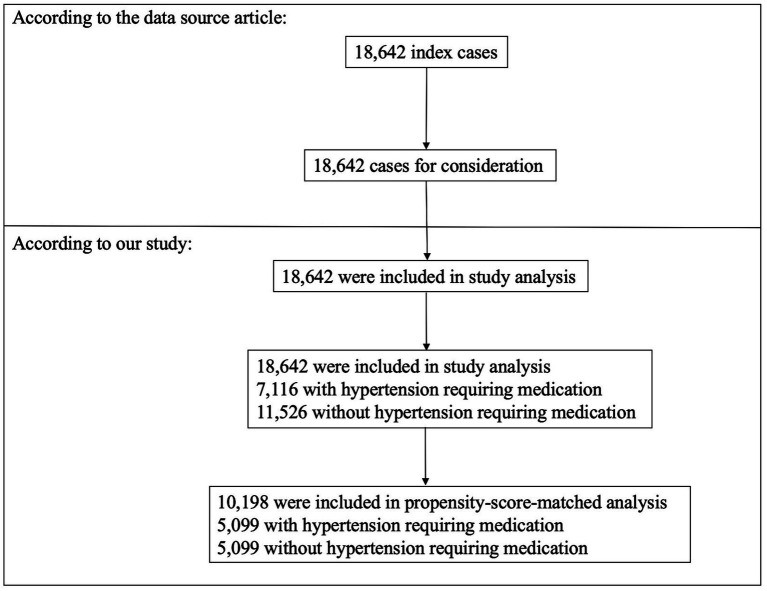
Flowchart of the study participants.

A total of 18,642 individuals met the inclusion criteria (Figure 1), 52.6% of whom were male and 47.4% of whom were female. This population included 7,116 (38.17%) patients with hypertension requiring medication and 11,526 (61.83%) patients without hypertension requiring medication. Prior to PSM, several fundamental attributes differed between individuals with hypertension requiring medication and without hypertension requiring medication (Table 1). We found that individuals with hypertension requiring medication generally had higher BMIs; Na, BUN, and Cr levels; and WBC counts. With the use of one-to-one PSM, 5,099 patients with hypertension requiring medication were matched with 5,099 patients without hypertension requiring medication. Following the matching process, the majority of variables (excluding race and diabetes status) exhibited standardized differences of less than 10.0%, suggesting successful matching of propensity scores. Specifically, fundamental attributes exhibited minimal disparities between the two cohorts.

### 3.2 Propensity score model

**Figure 2:**
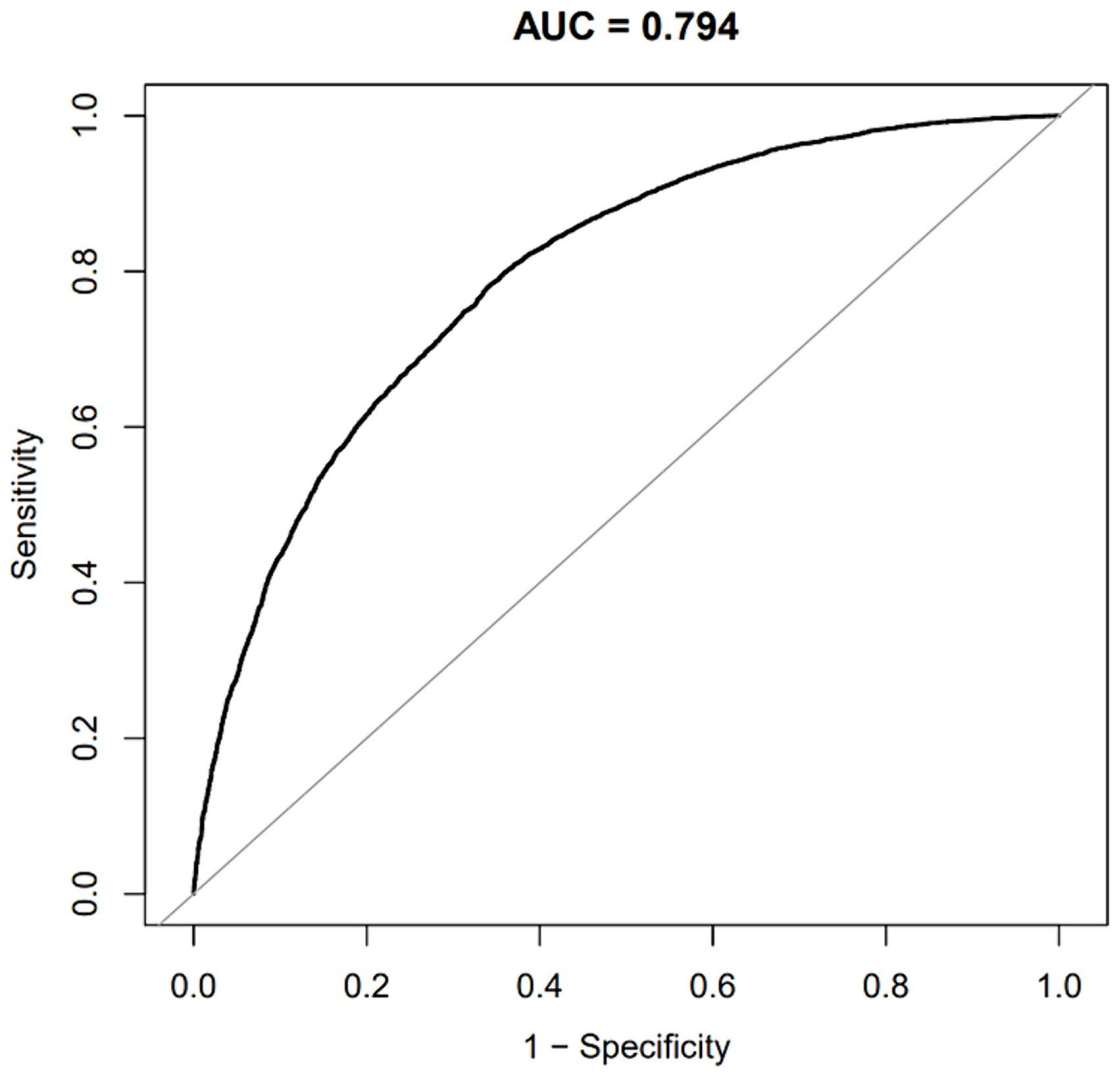
ROC curve of the propensity score for predicting hypertension requiring medication.

A logistic regression model was used to calculate the propensity score, resulting in a c-statistic of 0.794 (Figure 2).

### 3.3 Association between hypertension requiring medication and postoperative 30-day mortality

With respect to the propensity score-matched cohort, we employed a binary logistic regression model to assess the association between hypertension requiring medication and postoperative 30-day mortality. We present the findings from unadjusted, minimally adjusted, fully adjusted, and propensity score-adjusted analyses simultaneously (Table 2). According to the basic model, hypertension requiring medication was strongly associated with postoperative 30-day mortality (OR = 1.366, 95% CI 1.060–1.758; *p* = 0.0157). Specifically, individuals with hypertension requiring medication had a 36.6% greater chance of experiencing postoperative 30-day mortality than did those without hypertension requiring medication. With minimal adjustments (adjusted for sex, race, and age groups), the strong correlation remained significant (OR = 1.386, 95% CI 1.074–1.788, *p* = 0.0121). We were also able to identify a significant correlation after accounting for all the covariates (sex, ethnicity, age groups, body mass index, hematocrit, sodium, blood urea nitrogen, white blood cell count, creatinine, platelet count, duration of surgery, diabetes, smoking habits, year of surgery, functional health status, severe chronic obstructive pulmonary disease, congestive heart failure, presurgical transfusions, metastatic cancer, presurgical systemic sepsis, steroid use for chronic illness, more than 10% weight loss in the past 6 months, bleeding disorders, emergency surgery, and wound classification) (odds ratio = 1.390, 95% CI 1.071–1.804, *p* = 0.01324). After adjusting for propensity scores, the likelihood of postoperative 30-day mortality increased by 34% among individuals with hypertension requiring medication (odds ratio = 1.340, 95% CI 1.0401.727, *p* = 0.02366). These findings suggest that patients with hypertension requiring medication prior to craniotomy have an increased risk of postoperative 30-day mortality.

**Table 2:** Associations between hypertension requiring medication and postoperative 30-day mortality according to different models.

Variable	Crude model (OR,95% CI, P)	Model I (OR, 95% CI, P)	Model II (OR, 95% CI, P)	Model III (OR, 95% CI, P)
Nonhypertension	Ref.	Ref.	Ref.	Ref.
Hypertension	1.366 (1.060, 1.758) 0.01574	1.386 (1.074, 1.788) 0.01210	1.390 (1.071, 1.804) 0.01324	1.340 (1.040, 1.727) 0.02366

Crude model: We did not adjust for other covariates. Model I: We adjusted for sex, race, and age ranges. Model II: We adjusted for variables such as sex, ethnicity, age group, body mass index (BMI), hematocrit (HCT), sodium (Na), blood urea nitrogen (BUN), white blood cell count (WBC), creatinine (Cr), platelet count (PLT), duration of surgery, presence of diabetes, smoking habits, year of surgery, overall health condition, severe chronic obstructive pulmonary disease (COPD), congestive heart failure (CHF), presurgical blood transfusions, presence of disseminated cancer, presurgical systemic sepsis, use of steroids for chronic conditions, weight loss exceeding 10% in the past 6 months, bleeding disorders, emergency cases, and wound classification. In Model III, we adjusted for the propensity score. Hazard ratios (HRs), confidence intervals (CIs), and reference categories (Ref) are the terms used in this context.

### 3.4 Sensitivity analysis

We used the IPTW to generate a weighted cohort. To guarantee the strength of the findings, we conducted a binary logistic regression analysis to evaluate the association between hypertension requiring medication and postoperative 30-day mortality in both the initial group and the weighted group. Table 3 displays the models that were not adjusted, minimally adjusted, or fully adjusted for the two cohorts. In both the initial group and the weighted group, we discovered a correlation between postoperative 30-day mortality and the probability of postoperative 30-day mortality. According to the full model, patients with hypertension requiring medication had a 39.2% greater risk of postoperative 30-day mortality in the original cohort (OR = 1.392, 95% CI 1.120, 1.729, *p* = 0.00285) and a 37.6% greater risk in the weighted cohort (OR = 1.376, 95% CI 1.202, 1.576, *p* < 0.00001) than patients without hypertension requiring medication.

**Table 3:** Associations between hypertension requiring medication and postoperative 30-day mortality according to the original and weighted cohort (IPTW) models.

Variable		Crude model (OR, 95% CI, P)	Model I (OR, 95% CI, P)	Model II (OR, 95% CI, P)
A	Nonhypertension	Ref.	Ref.	Ref.
	Hypertension	2.292 (1.899, 2.766) <0.00001	1.582 (1.292, 1.937) <0.00001	1.392 (1.120, 1.729) 0.00285
B	Nonhypertension	Ref.	Ref.	Ref.
	Hypertension	0.936 (0.831, 1.053) 0.26966	1.066 (0.942, 1.206) 0.31025	1.376 (1.202, 1.576) <0.00001

A, In the original cohort; B, In the weighted cohort (IPTW). Crude model: We did not modify any other variables. Model I: We adjusted for sex, race, and age range. Model II: We adjusted for variables such as sex, ethnicity, age group, body mass index (BMI), hematocrit (HCT), sodium (Na), blood urea nitrogen (BUN), white blood cell count (WBC), creatinine (Cr), platelet count (PLT), duration of surgery, presence of diabetes, smoking habits, year of surgery, overall health condition, severe chronic obstructive pulmonary disease (COPD), congestive heart failure (CHF), presurgical blood transfusions, presence of disseminated cancer, presurgical systemic sepsis, use of steroids for chronic conditions, weight loss exceeding 10% in the past 6 months, bleeding disorders, emergency cases, and wound classification. Hazard ratios (HRs), confidence intervals (CIs), and reference categories (Ref) are the terms used in this context.

In addition, the authors generated an E value to assess the sensitivity to unmeasured confounding variables. The value of E was 2.13. The E value exceeded the relative risk associated with unmeasured confounders and hypertension requiring medication, indicating that unmeasured or unidentified confounders had minimal impact on the association between hypertension necessitating medication and postoperative 30-day mortality.

## 4 Discussion

In current studies on various types of brain tumors, such as brain metastasis, meningioma, and glioblastoma (18, 27–30), PSM is commonly utilized to account for variations in baseline characteristics between two groups. After matching to balance the baseline characteristics of 16,335 participants in the 2012–2018 NSQIP database, the duration of surgery was shown to predict the prognosis following craniotomy for supratentorial brain tumors (29). Similarly, we adopted the PSM method to ensure homogeneity in the participants’ backgrounds. By utilizing information on hypertension requiring medication and postoperative 30-day mortality from the ACS NSQIP database, we discovered that employing three distinct approaches to manage potential bias did not yield significant disparities in the estimated impact, with odds ratios ranging from 1.340 (95% CI 1.040, 1.727) for the propensity score-matched analysis to 1.390 (95% CI 1.071, 1.804) after accounting for all covariates. This retrospective study of individualized PSMs revealed that hypertension requiring medication was a major factor influencing postoperative 30-day mortality after craniotomy for tumor resection in adult patients in the U.S., with odds ratios ranging from 1.34 to 1.39. Gaining comprehensive knowledge about hypertension requiring medication as a potential determinant of postoperative 30-day mortality will enhance our comprehension and communication of risks with patients, ultimately resulting in the development of more individualized prevention and management strategies.

Several prior investigations have indicated that high blood pressure is linked to hidden brain conditions, including cognitive Decline, cerebrovascular accidents, and subclinical abnormalities in the blood vessels of the brain (31–35). Hypertension causes alterations in cerebral small- and medium-to-large artery function and structure that can impair blood flow and are related to oxidative stress, endothelial dysfunction and inflammation (32–34). Dolui S et al. confirmed that intensive blood pressure treatment, compared with standard treatment, was associated with an increase, not a Decrease, in cerebral perfusion and was most notably associated with a history of cardiovascular disease (36). Severe perioperative hypertension can result in excessive surgical bleeding, which May increase mortality (12, 15). Dasenbrock et al. proposed that high blood pressure was a factor that could predict the need for a second surgery to remove a blood clot after skull surgery for a tumor, and the removal of the blood clot was strongly linked to a greater risk of death within 30 days after the operation (12). According to the aforementioned research, changes in the structure of the cerebral artery and cerebral blood flow, the occurrence of cerebral hemorrhage after surgery, and the presence of other cardiovascular ailments might significantly contribute to the 30-day mortality rate in brain tumor patients with hypertension requiring medication. Villela PB et al. reported that deaths associated with hypertensive diseases were mentioned up to 4 times more frequently in Brazil from 2004 to 2013 when evaluated as multiple causes of death rather than when selected as the primary cause of death. This finding highlights the critical importance of improving hypertension control to prevent fatalities (37).

Our research has certain notable advantages that should be acknowledged. To our knowledge, few retrospective investigations have utilized PSM to examine the correlation between hypertension requiring medication and 30-day mortality following craniotomy for tumor resection in adult patients. PSM aims to equalize the allocation of recorded baseline covariates to reduce the impact of observed confounding variables. To ensure the dependability of the findings, a sensitivity analysis was performed, specifically utilizing the IPTW method to create a weighted group. The relationship between hypertension requiring medication and postoperative 30-day mortality was subsequently investigated in this weighted cohort. Furthermore, compared with the majority of prior analogous investigations, our sample size was relatively large, and the participants were recruited from various centers.

Conversely, our study has several limitations that should be noted. First, because our study relies on a secondary examination of published information, we were unable to completely eliminate certain residual and/or unmeasured confounding variables that May influence the estimated correlation (for instance, various factors, including eating patterns, economic conditions, medication therapies, properties of noncancerous and cancerous tissues, and diverse categories and locations of brain tumors). The database used in our study lacked information on pharmacological treatments for hypertension. Nevertheless, we calculated the E value to assess the possible impact of unmeasured variables. The study utilized data gathered from a diverse and sizable population of individuals diagnosed with brain tumors. Therefore, the association and our other findings remain highly plausible. Further extensive investigations should gather and assess the effects of the aforementioned potential variables and information that were not incorporated in the current research.

## 5 Conclusion

This retrospective study of individualized PSMs revealed that hypertension requiring medication was a major factor influencing 30-day mortality after craniotomy for tumor resection in adult patients in the United States, with odds ratios ranging from 1.34 to 1.39. Among patients who required medication for hypertension and had high propensity scores, the risk of postoperative 30-day mortality increased by 34–39% compared with patients without hypertension requiring medication and had low propensity scores. In adult patients undergoing craniotomy for tumor resection, blood pressure is a risk factor that can be modified through interventions to prevent postoperative 30-day mortality. For patients with hypertension requiring medication, preoperative blood pressure should be controlled by drugs before craniotomy to reduce the risk of postoperative complications.

## Data Availability

The original contributions presented in the study are included in the article/Supplementary material, further inquiries can be directed to the corresponding author/s.

## Footnotes

^1^http://www.R-project.org, The R Foundation.
